# Psychological effect of comprehensive nursing intervention in elderly patients with perforated peptic ulcer: A protocol of systematic review

**DOI:** 10.1097/MD.0000000000022226

**Published:** 2020-09-25

**Authors:** Bing Chen, Xiu-Yu Liu, Hong-Mei Zhang, Bai-Jun Zhang, Ying-Ting Wang

**Affiliations:** a Department of Gastroenterology, The Second Affiliated Hospital of Mudanjiang Medical University; b Department of Emergency, Mudanjiang Forestry Central Hospital; c Department of Geriatrics, The Second Affiliated Hospital of Mudanjiang Medical University, Mudanjiang, China.

**Keywords:** anxiety, comprehensive nursing intervention, depression, perforated peptic ulcer

## Abstract

**Background:**

This study aims to assess the psychological effect of comprehensive nursing intervention (CNI) in elderly patients with perforated peptic ulcer (PPU).

**Methods:**

This protocol will search all potential studies from inception to the present in electronic database sources (Cochrane Library, PUBMED, EMBASE, PsycINFO, WANGFANG, CBM, and CNKI), and other sources (such as clinical trial registry, and conference proceedings). We will not apply limitations to language and publication status. Two independent authors will scan literature, extract data, and appraise study quality. A third author will be invited to solve any disagreements between 2 authors. We will utilize RevMan 5.3 software for statistical analysis. If necessary, we will also carry out subgroup group, sensitivity analysis, and reporting bias.

**Results:**

This protocol will summarize high quality evidence to evaluate the psychological effect of CNI in elderly patients with PPU.

**Conclusion:**

The results of this study may provide evidence to determine whether CNI is effective or not on psychological effect in elderly patients with PPU.

**Study registration:**

INPLASY202080069.

## 1 Introduction

Peptic ulcer disease (PUD) is a very common gastrointestinal disease, which usually occurs in the stomach and proximal duodenum.^[1–3]^ It symptoms and signs mainly manifest as gnawing or burning pain in middle or upper stomach, bloating, heartburn, and nausea or vomiting.^[4,5]^ Risk factors are responsible for such disorder, such as helicobacter pylori bacteria, frequent use of nonsteroidal anti-inflammatory drugs, and family history of PUD.^[6–8]^ It is reported that its prevalence rate ranges from 5% to 12% worldwide.^[3,9–11]^ If it cannot be treated timely and effectively, it may result in several complications, such as perforated peptic ulcer (PPU), gastrointestinal bleeding, gastric outlet obstruction, penetration, and even gastric cancer.^[12–17]^ Of those, PPU accounts for about 2% to 10% of all patients with PUD.^[18]^ Thus, it is very important to detect and treat PPU at early stage. Surgery is the most effective management for PPU.^[19,20]^ However, most patients with PPU also suffer from psychological disorder (including depression and anxiety).^[21]^ Previous studies have reported that comprehensive nursing intervention (CNI) can be utilized for the management of elderly patients with PPU.^[22–28]^ However, there are inconsistent results, and no systematic review has investigated the effects of CNI on psychological disorder in elderly patients with PPU. Thus, this study will systematically and comprehensively assess the psychological effect of CNI in elderly patients with PPU.

## 2 Methods

### 2.1 Study registration

We have registered this study protocol on INPLASY202080069, and we report it according to the guidelines of the Preferred Reporting Items for Systematic Reviews and Meta-Analysis Protocol statement guidelines.^[29,30]^

### 2.2 Eligibility criteria

#### 2.2.1 Types of studies

This study will include randomized controlled trials of CNI on psychological effect in elderly patients with PPU. We will eliminate other studies, such as nonclinical trial, and uncontrolled trial.

#### 2.2.2 Types of participants

Elderly patients (over 65 years old) with PPU who were also diagnosed as psychological disorder (including depression and anxiety) will be included, regardless gender, severity of psychological condition, and PPU.

#### 2.2.3 Types of interventions

In the intervention group, all eligible patients administered CNI on psychological disorder.

In the control group, all patients underwent other managements will be included. However, we will exclude comparator involving any forms of CNI.

#### 2.2.4 Type of outcome measurements

Primary outcome is psychological disorder. It comprises of depression and anxiety, as measured by Beck Depression Inventory and Hamilton Depression Rating Scale, or other relevant scales.

Secondary outcomes are health-related quality of life (as assessed by Global Quality of Life Scale), panic (as examined by Panic Disorder Severity Scale), and adverse events.

### 2.3 Literature sources

#### 2.3.1 Electronic database sources

We will retrieve all potential studies from inception to the present in the Cochrane Library, PUBMED, EMBASE, PsycINFO, WANGFANG, CBM, and CNKI. No restrictions will be employed to the language and publication status. We summarize the sample of search strategy for PUBMED in Table 1. We will also modify similar search strategies for other electronic databases.

**Table 1:**
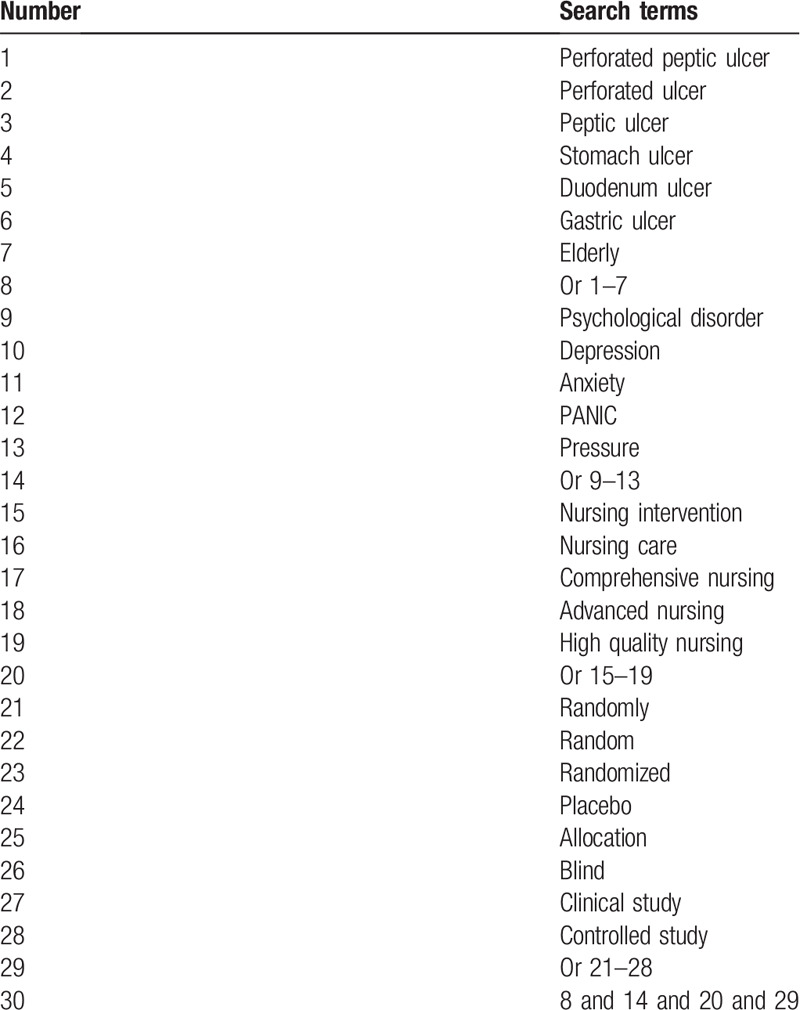
Detailed search strategy of PUBMED.

#### 2.3.2 Other sources

We will search other sources to avoid missing potential studies, such as clinical trial registry, conference proceedings, and reference list of included studies.

### 2.4 Data collection and analysis

#### 2.4.1 Study selection

Two independent authors will scan titles/abstracts of all searched studies and all irrelevant literature will be eliminated. Then, full text of potential articles will be cautiously read in accordance with all inclusion criteria. All eligible studies will be included in this study, and all excluded studies will be recorded with reasons. Any disagreements will be solved by a third author through discussion. We will summarize the process of study selection in a flow diagram.

#### 2.4.2 Data extraction

Two authors will independently extract data according to the previously designed data extraction form. It includes publication characteristics (such as title, first author, journal, and study design), patient characteristics (such as number of patients, age, gender, diagnosis criteria, and inclusion and exclusion criteria), study methods, details of CNI and controls, outcome indicators, results, conclusion, and follow-up information. Any divergences will be resolved by a third author via discussion.

#### 2.4.3 Study quality assessment

Two authors will independently examine methodological quality of each included study using Cochrane Risk of Bias Tool. We will invite a third author to clear any confusion between 2 authors.

#### 2.4.4 Dealing with missing data

If any insufficient or missing information occurs, we will contact original study authors to obtain it by email or fax. If it is not available, we will only analyze available data.

#### 2.4.5 Data synthesis

We will use RevMan 5.3 software to conduct statistical analysis. For dichotomous data, we will calculate it as risk ratio and 95% confidence intervals. For continuous data,

We will estimate it as weighted mean difference or standardized mean difference and 95% confidence intervals.

Statistical heterogeneity will be identified by *I*^2^ test. Values of *I*^2^ illustrate as follows: *I*^2^ ≤ 50% means reasonable heterogeneity, and we will use a fixed-effects model to integrate outcome data. *I*^2^ > 50% signifies a substantial heterogeneity, and we will employ a random-effects model to combine outcome data. If the extracted data similar sufficiently on the same outcome measurement, we will synthesize those data and will carry out a meta-analysis. If there is remarkable heterogeneity across included studies, we will conduct a qualitative synthesis using narrative summary descriptions. In addition, we will undertake subgroup and sensitivity analysis to investigate the possible reasons of obvious heterogeneity.

#### 2.4.6 Reporting bias

Any possible reporting bias will be checked using Funnel plot and Egger regression test when over 10 studies are eligible.^[31,32]^

#### 2.4.7 Subgroup analysis

We will conduct subgroup analysis to test the sources of significant heterogeneity based on characteristics of study, severity of psychological disorder or PPU, and details of CNI and controls.

#### 2.4.8 Sensitivity analysis

We will perform sensitivity analysis to test robustness and stability of the present results by removing studies with low quality and small sample size.

### 2.5 Dissemination and ethics

We plan to publish this study on a peer-reviewed journal. This study does not need ethical approval, because it will only extract data from the exist studies.

## 3 Discussion

According to the best of our knowledge, this systematic review is the first one to examine the psychological effect of CNI in elderly patients with PPU. Although previous studies suggested utilizing CNI for the management of psychological disorder in elderly patients with PPU, their results were controversial up to now. In addition, there were no consensus and existing recommendations of CNI on psychological disorders in elderly patients with PPU specifically.

Therefore, considering this urgent demand, we will organize this systematic review through performing comprehensive literature search, and rigorous evidence synthesis. We also registered this protocol to make sure it is transparent. We will ensure that the findings of this study will provide rigorous evidence regarding whether CNI is effective or not on psychological disorder in elderly patients with PPU. It may also benefit both patients and clinicians.

## Author contributions

**Conceptualization:** Bing Chen, Xiu-Yu Liu, Hong-Mei Zhang, Bai-Jun Zhang.

**Data curation:** Bing Chen, Xiu-Yu Liu, Ying-Ting Wang.

**Formal analysis:** Bing Chen, Xiu-Yu Liu.

**Investigation:** Ying-Ting Wang.

**Methodology:** Bing Chen, Hong-Mei Zhang, Bai-Jun Zhang.

**Project administration:** Ying-Ting Wang.

**Resources:** Bing Chen, Xiu-Yu Liu, Hong-Mei Zhang, Bai-Jun Zhang.

**Software:** Bing Chen, Xiu-Yu Liu, Hong-Mei Zhang, Bai-Jun Zhang.

**Supervision:** Ying-Ting Wang.

**Validation:** Bing Chen, Xiu-Yu Liu, Hong-Mei Zhang, Bai-Jun Zhang, Ying-Ting Wang.

**Visualization:** Bing Chen, Bai-Jun Zhang, Ying-Ting Wang.

**Writing – original draft:** Bing Chen, Xiu-Yu Liu, Hong-Mei Zhang, Bai-Jun Zhang, Ying-Ting Wang.

**Writing – review & editing:** Bing Chen, Bai-Jun Zhang, Ying-Ting Wang.
